# Suggestive Therapeutics—A Treatise on Nature and Uses of Hypnotism

**Published:** 1889-05

**Authors:** 


**Suggestive Therapeutics. A Treatise on the Nature and Uses of Hypnotism.** By H. BERNHEIM, M. D., Professor in the Faculty of Medicine at Nancy. Translated from the second and revised French edition by Christian A. Herter, M. D., of New York. New York and London: G. P. Putnam's Sons. The Knickerbocker Press. 1889.

Prof. Bernheim has been very fair in his treatment of his rivals and opponents, quoting their criticisms of his work at great length, and replying to them with more than ordinary vigor.

As is well known, Charcot and Bernheim are at variance as to the theory of hypnotism and as to its uses. Charcot holds that it is a pathological state, and is only completely obtained in hysterical persons. Bernheim, on the contrary, says, page 8, in his preface: "No; hypnotic sleep is not a pathological sleep. The hypnotic condition is not a neurosis." The translator's preface closes with this sentence: "What I do claim for it, is that, being based upon the conscientious and well-directed study of an enormous number of cases by an able clinician, it may justly be considered the most scientific and important contribution to our knowledge of hypnotism that has yet been made." This opinion by an enthusiastic follower of Bernheim is, in the main, correct. For the first time, we have in a single volume a complete discussion of an imperfectly understood subject analogous to hysteria. He says also: "There is nothing by which to differentiate this induced sleep from natural sleep."

He develops, with great ability, the idea that hypnotic sleep is itself the result of a suggestion, and that all the phenomena which occur in that state are the result of suggestion. Few readers will disagree with this: hypnotism *is* the result of suggestion. But few of us, however, will accept his statement that it cannot be differentiated from natural sleep.

He asserts that he has been able to produce the same phenomena in persons who have gone to sleep naturally, by putting himself into relation with them and suggesting various ideas.

When we read this volume, and note the marvelous performances with which his patients enliven his clinic, we are obliged to say that Americans, at any rate, do not sleep that kind of sleep. If pins are stuck into us, we awaken; we are not anesthetic merely because we are told so. It seems to us that he has proved his point that hypnotism is the result of suggestion, but that he has not proven successfully that it is a natural sleep. He claims that five-sixths of all persons are hypnotizable. This is a very large percentage—much larger than has been obtained in other countries. Contrary to many authorities, he asserts that, when well managed, it does not produce the slightest harm. Meinert, on the contrary, prohibits hypnotism in his wards, believing that the physician has no right to induce a diseased condition. In the first five chapters we are given his rules for hypnotizing, and a classification of the different degrees of hypnotism, from slight drowsiness to somnambulism.

The last portion of the book is clinical. One hundred and five cases are reported. We notice such cases as the following: Case of cerebral hemorrhage, hemiplegia hemianesthesia, with tremor and contracture; cure. Also cures of rebellious sciatica, and cures of amenorrhea and menorrhagia by the use of suggestive treatment only.

The majority of cases reported as cured are purely nervous functional diseases, and with these we can take no exception. But to be asked to believe that the disorders of menstruation can be treated by suggestion, is asking for more faith than is possessed by most of us. Two cases are reported—one a case of amenorrhea, in which the flow was restored by suggestion. The other was a case of menorrhagia; the flow was controlled by suggestion. We cannot, on the strength of these two cases, believe that in this mysterious therapeutic agent there is a practical means of treating menstrual diseases.

The work, however, is eminently scientific in its treatment ot the subject in hand, and the author has, we think, given us a correct explanation of many of the phenomena of mind-healing faith-cure metallotherapy. Bernheim deserves great credit for stripping the mask of mystery from these uncanny cases, and presenting his views in so clear a form.

The book is a handsome octavo, bound in cloth. Its clear type and good paper add a pleasure to the profit that one gets from its perusal.

